# Treatment of Hospital Gangrene

**Published:** 1864-09

**Authors:** Hargrove Hinkley

**Affiliations:** Demopolis, Ala.


ART. II.—*Treatment of Hospital Gangrene.* By Surgeon HARGROVE HINKLEY, of Demopolis, Ala.

The success I have met with in the treatment of hospital gangrene, induces me to send you some notes from my case-book regarding it. When I took charge of the hospital at this place, there were several very bad cases in the surgical ward, which had progressed unfavorably, in spite of treatment. I copy from my case-book:

Case 1.—J. Cope, sergeant of company "K," 18th Mississippi volunteers; wounded at Gettysburg, May 3d, 1863; flesh wound in right arm, anterior to bone. Patient walked two hundred miles after receiving wound. Sent to hospital at Richmond. Wound healed over, and patient left hospital on furlough for Mississippi. Arrived at Demopolis, Alabama, last July, with violent fever; remained in quartermaster's warehouse one day, and was then moved to this hospital, at that time in charge of Surgeon Somerville Burke. Erysipelatous inflammation attacked the wound in a day or two, and gangrene supervened. The disease advanced rapidly, bidding defiance to the treatment used. On the 22d of August, when I took charge, the muscles and tissues on the anterior and lateral portions of the arm were gone, and the bone and arteries visible, but a small portion of healthy flesh remaining posteriorly. The space already destroyed involved the middle-third of the arm anteriorly and laterally, and the muscles were dissected by the gangrene to above insertion of deltoid and nearly to elbow. The treatment hitherto pursued not having had any effect, and the disease advancing rapidly, I decided, two or three days after taking charge, to amputate at the shoulder-joint, as a *dernier* resort, with but faint hope of saving life. The condition of the patient was bad in the extreme for an operation. Wasted to a skeleton; pulse low and frequent; cold, clammy perspiration; suffering intensely and well-nigh exhausted; the general opinion of all the medical men who saw the case before operating was, the patient would not survive the operation.

On the 26th of August, the patient being well stimulated and put under the influence of chloroform on the operating table, placed out of doors in the open air, I amputated at the shoulder-joint. The blood-vessels were somewhat enlarged, and some redness was observed on the flaps. Patient recovered from the influence of chloroform without any bad result and with much moral courage, and expressed hope and confidence in the attendants. Pulse, four or five hours after operation, much improved in volume, and patient in better condition than before the operation. Wound dressed next day, and to all appearance doing well. Patient expressed himself better, and partook freely of nourishing food and stimulants. Ordered muriated tincture of iron, twenty drops, three times a day; opium, one grain, three times a day.

On the fourth day after the operation, symptoms of gangrene appeared, and in a few days the flaps had sloughed away and some adjacent tissues. Patient hopeful and placing implicit reliance on the efforts made to save him. The treatment adopted consisted in stimulants, *i. e.* brandy-toddy and egg-nogg, *ad libitum.* The most nourishing food consisting of soups, roast fowl, eggs, bread and butter, milk and coffee. Tincture of iron, gradually increased to twenty-five drops, three times a day; opium, three to four grains a day; nitric acid to slough; the wound well washed out with soap and water; dead parts removed, and freely washed off with turpentine and Labarraque's solution of chlor. soda; tincture of iodine painted on edges and around the wound; tincture of iron applied once to wound itself; slough checked in five or six days, and granulations of a healthy character made their appearance and advanced so rapidly, that, by the nineteenth day after the operation, the granulations had filled the wound up even with the surrounding sound parts. Cicatrization rapidly followed. Bed sores, which had appeared and been attacked by gangrene, were subjected to same local treatment and relieved by circular (ring) cushions, and though somewhat obstinate, yet gradually yielded to treatment. Patient entirely recovered by the first week in November.

Case 2.—C. W. Fitzpatrick, corporal of company "D," 6th Missouri volunteers, admitted to this hospital July 9th, 1863. Wounded May 16th, 1863, at Baker's creek; injuring the phalangeal and metatarsal articulation of great toe of right foot; missile a minie ball. Moved from battle-field to Clinton, thence to this hospital, when erysipelatous inflammation took place, gangrene supervening and resulting in the loss of large and second toe. Gangrene checked, August 30th, by local applications of nitric acid, turpentine and chlor. soda, (Labarraque's solution,) and tinctura ferri sesquichlor. and opium internally. Stimulants, *ad libitum*, and nourishing diet. December 31st, healed over.

Case 3.—E. D. Wilson, private, company "I," 27th Louisiana volunteers; wounded at Chickamauga, on the 20th day of September, 1863. Flesh wound of left leg in middle-third, posterior surface, severe; missile a minie ball. Compound fracture of the tibia of right leg, middle-third; ligation of tibial artery. September 21st. Application of splints; cold-water dressing to both wounds. Left Atlanta on the 21st of November; passed through here to Meridian, and was sent back here for treatment by Dr. Scott, Medical Director; reached here on the 29th December, 1863. Condition of wounds bad; extensive sloughing having taken place in right leg, and wounds presenting a very serious aspect.

*Treatment.*—Stimulants, nourishing diet, and tincture iron and opium. *Local Treatment.*—Nitric acid, sol. chlor. soda and turpentine. Under this treatment, the condition of the wounds rapidly improved, and, on January 11th, the case was convalescent.

Case 4.—Sidney Anderson, private company "B," 20th Alabama; admitted December 8th, 1863; wounded at Chickamauga by a grape-shot entering anterior portion of upper-third of thigh, fracturing femur. Amputation performed just above the wound on September 22d. Favorable progress until wound was nearly cicatrized. Left Fair Ground Hospital, Atlanta, on December 30th, 1863. On arrival at Selma Wayside Hospital, erysipelatous inflammation had taken place, and, when he arrived here, gangrene had supervened. Ordered muriated tinct. iron, fifteen drops, three times a day; brandy-toddy through day and night; diet full and nourishing; wound dressed with Labarraque's sol. chlor. soda.

December 9th. Treatment continued; opium, one grain, at night. Dec. 10th. Wound freely touched with nitric acid; treatment continued. Dec. 11th. Slough increasing; treatment continued. Dec. 12th. Nitric acid again applied, and flaps sloughed off; gangrene burrowing in dissecting muscles; treatment continued. Ordered opium, one grain, ter in die. Sound parts painted with tinct. iodine. Dec. 13th. Slough checked above; still progressing on under and inner portion. Nitric acid applied to these parts. General treatment continued. Opium changed to sulph. morphia, half grain, ter in die; to be taken half an hour before dressing wound and at 10 P. M. Patient's condition bad; pulse feeble and frequent; much despondency; bowels tending to diarrhœa; countenance haggard. The sloughing has left no place to operate but at hip-joint, which, in this case, must prove fatal. Gangrene must be checked or patient succumb. Dec. 14th. Treatment continued; condition same. Quite a pocket exists on under side, the muscles being disorganized up to two or three inches of the groin, the skin being left. Care taken after dressing the wound with turpentine and sol. chlor. soda; to dry it well with lint before closing it. Dec. 15th. Treatment continued; slough checked; healthy granulations on all parts, except inner part of thigh. Dec. 16th. Patient improving; pulse and strength better; healthy granulations all over. Dec. 17th. Treatment continued; decided improvement. December 31st. Patient has been kept on same treatment—iron, morphine, stimulants and nourishing diet, and has much improved. Small slough appeared yesterday, but is gone to-day. Strength improving, and granulations rapidly forming. During all this time the patient has been by himself in a tent. Being much improved, and the weather very cold, he was removed into a ward, one hundred feet long and thirty feet wide, sixteen feet high to collar beams, with no ceiling between roof and floor, and having three ventilators open in roof and fifteen windows in the ward. It is warmed by three small wood stoves. He was now taken charge of by one of my assistants, Dr. John E. Pugh, myself still having general supervision.

January 1st, 1864. Wound not quite so well to-day; some little sloughing apparent. Nitric acid touched to several spots; opium omitted. Jan. 2d. Stump healthier; treatment continued. Jan. 3d. Patient restless; pulse small; stump sloughing at lower edge; touched with nitric acid. Ordered sulph. quinine, grs. xij; opii, grs. ix. M. ft. pil. 9. S. take one three times a day. Also sulph: morph, gr. ¼, at 11 P. M. Other treatment continued. Jan. 4th. Continued treatment. Jan. 5th. Patient slept well; pulse feeble; appetite poor. Jan. 6th. Condition unchanged; treatment continued. Jan. 7th. Ditto of yesterday. Jan. 8th. Stump improving; treatment continued. Jan. 14th. Patient continued to improve daily, and the stump to show healthy granulations all over; filling up rapidly; the treatment continued. January 15th. Patient doing well; no appearance of gangrene. Jan. 22d. Patient has improved very rapidly the past week, and is getting stronger; wound closing fast; granulations entirely healthy, and bids fair to get entirely well now.

There were four other cases of gangrene, not so serious as those above-mentioned, and which all yielded to the treatment adopted. When the tincture of iron was suspended for a few days, the gangrene would re-appear or grow worse, and when the opium was diminished, the case would assume an unfavorable aspect. Muriated tincture of iron and opium internally, with brandy or whiskey-toddy, egg-nogg, and rich food, with local treatment of nitric acid, turpentine and sol. chlor. soda, (Labarraque's,) constitute the main treatment. Patients to have plenty of fresh air. I have not lost a case of gangrene during the war which has been treated as above, and have seen but one death as yet from this disease.

